# Approximation solution for system of generalized ordered variational inclusions with ⊕ operator in ordered Banach space

**DOI:** 10.1186/s13660-017-1351-x

**Published:** 2017-04-20

**Authors:** Mohd. Sarfaraz, MK Ahmad, A Kılıçman

**Affiliations:** 1 Department of Mathematics, Aligarh Muslim University, Aligarh, 202002 India; 2 Department of Mathematics, University Putra Malaysia (UPM), Serdang, Selangor 43400 Malaysia

**Keywords:** 49J40, 47H05, algorithm, convergence, resolvent, solution, system, ordered Banach space

## Abstract

The resolvent operator approach is applied to address a system of generalized ordered variational inclusions with ⊕ operator in real ordered Banach space. With the help of the resolvent operator technique, Li *et al.* (J. Inequal. Appl. 2013:514, 2013; Fixed Point Theory Appl. 2014:122, 2014; Fixed Point Theory Appl. 2014:146, 2014; Appl. Math. Lett. 25:1384-1388, 2012; Fixed Point Theory Appl. 2013:241, 2013; Eur. J. Oper. Res. 16(1):1-8, 2011; Fixed Point Theory Appl. 2014:79, 2014; Nonlinear Anal. Forum 13(2):205-214, 2008; Nonlinear Anal. Forum 14: 89-97, 2009) derived an iterative algorithm for approximating a solution of the considered system. Here, we prove an existence result for the solution of the system of generalized ordered variational inclusions and deal with a convergence scheme for the algorithms under some appropriate conditions. Some special cases are also discussed.

## Introduction

The theory of variational inequalities (inclusions) is quite application oriented and thus developed much in recent years in many different disciplines. This theory provides us with a framework to understand and solve many problems arising in the field of economics, optimization, transportation, elasticity and applied sciences. A lot of work considered with the ordered variational inequalities and ordered equations was done by Li *et al.*; see [4, 6, 8, 9].

The fundamental goal in the theory of variational inequality is to develop a streamline algorithm for solving a variational inequality and its various forms. These methods include the projection method and its novel forms, approximation techniques, Newton’s methods and the methods derived from the auxiliary principle techniques.

It is widely known that the projection technique cannot be applied to solve variational inclusion problems and thus one has to use resolvent operator techniques to solve them. The beauty of the iterative methods involving the resolvent operator is that the resolvent step involves the maximal monotone operator only, while other parts facilitate the problem’s decomposition. Most of the problems related to variational inclusions are solved by maximal monotone operators and their generalizations such as *H*-accretivity [10], *H*-monotonicity [11] and many more; see *e.g.*, [12–17] and the references therein.

Essentially, using the resolvent technique, one can show that the variational inclusions are commensurate to the fixed point problems. This equivalent formation has played a great job in designing some exotic techniques for solving variational inclusions and related optimization problems.

We initiate a study of a system of generalized ordered variational inclusions in real ordered Banach space. We design an iterative algorithm based on the resolvent operator for solving system of generalized ordered variational inclusions. We prove an existence as well as a convergence result for our problem. For more details of related work, we refer to [2, 18] and the references therein.

## Prelude

In the paper, assume that *X* be a real ordered Banach space endowed with norm $\|\cdot\|$, an inner product $\langle\cdot, \cdot\rangle$, a zero element *θ* and partial order ≤ defined by the normal cone *C* with a normal constant $\lambda_{C}$. The greatest lower bound and least upper bound for the set $\{p, q\}$ with partial order relation ≤ are denoted by $\operatorname{glb}\{p, q\}$ and $\operatorname{lub}\{p, q\}$, respectively. Assume that $\operatorname{glb}\{p, q\}$ and $\operatorname{lub}\{p, q\} $ both exist.

The following well-known definitions and results are essential to achieve the goal of this paper.

### Definition 2.1

Let *C* (≠*ϕ*) be a closed, convex subset of *X*. *C* is said to be a cone if

(i) for $p\in C$ and $\lambda>0$, $\lambda p \in C$;
(ii) if *p* and $-p\in C$, then $p=\theta$.

### Definition 2.2

[19]

*C* is called a normal cone iff there exists a constant $\lambda_{C} >0$ such that $0\leq p \leq q$ implies $\|p\| \leq\lambda_{C} \|q\|$, where $\lambda _{C}$ is called the normal constant of *C*.

### Definition 2.3

For arbitrary elements $p,q\in X$, $p\leq q$ iff $p-q \in C$, then the relation ≤ is a partial ordered relation in *X*. The real Banach space *X* endowed with the ordered relation ≤ defined by *C* is called an ordered real Banach space.

### Definition 2.4

[20]

For arbitrary elements $p,q \in X$, if $p \leq q$ (or $q \leq p$) holds, then *p* and *q* are called comparable to each other and this is denoted by $p\propto q$.

### Definition 2.5

[18]

A map $A: X\rightarrow X$ is called a *β*-ordered comparison map, if it is a comparison mapping and

$$A(p)\oplus A(q) \leq\beta(p\oplus q),\quad \mbox{for } 0 < \beta< 1. $$

### Lemma 2.1

[19]

*If* *p* *and* *q* *are comparable to each other*, *then* $\operatorname{lub}\{p, q\}$ *and* $\operatorname{glb}\{p , q\}$ *exist*, $p-q \propto q-p$, *and* $\theta\leq(p-q) \vee(q-p)$.

### Lemma 2.2

[19]

*Let* *C* *be a normal cone with normal constant* $\lambda_{C}$ *in* *X*, *then for each* $p,q \in X$, *we have the relations*:

(i) $\|\theta\oplus\theta\|= \|\theta\|=\theta$;
(ii) $\|p \wedge q\| \leq\|p\| \wedge\|q\| \leq\|p\|+\|q\|$;
(iii) $\|p \oplus q\| \leq\|p-q\| \leq\lambda_{C} \|p \oplus q\|$;
(iv) *if* $p\propto q$, *then* $\|p\oplus q\|=\|p-q\|$.

### Lemma 2.3

[1, 4–6]

*Let* ≤ *be a partial order relation defined by the cone* *C* *with a normal constant* $\lambda_{C}$ *in* *X* *in Definition* 2.3. *Then hereinafter relations survive*:

1. $p \oplus q = q \oplus p$, $p\oplus p = \theta$;
2. $\theta\leq p \oplus\theta$;
3. *allow* *λ* *to be real*, *then* $(\lambda p)\oplus(\lambda q) = |\lambda| (p \oplus q)$;
4. *if* *p*, *q* *and* *w* *can be comparative to each other*, *then* $(p \oplus q) \leq(p\oplus w) + (w \oplus q)$;
5. *presume* $(p+q)\vee(s+t)$ *exists*, *and if* $p\propto s, t$ *and* $q \propto s,t$, *then* $(p+q) \oplus(s+t) \leq(p\oplus s + q \oplus t)\wedge(p\oplus t + q \oplus s)$;
6. *if* *p*, *q*, *r*, *w* *can be compared with each other*, *then* $(p\wedge q)\oplus(r \wedge w) \leq((p\oplus r)\vee(q \oplus w))\wedge((p\oplus w)\vee(q \oplus r))$;
7. *if* $p\leq q$ *and* $s\leq t$, *then* $p+s \leq q+t$;
8. *if* $p \propto\theta$, *then* $-p \oplus\theta\leq p \leq p\oplus\theta$;
9. *if* $p \propto q$, *then* $(p \oplus\theta)\oplus(q \oplus \theta) \leq(p\oplus q)\oplus\theta = p \oplus q$;
10. $(p\oplus\theta)- (q \oplus\theta) \leq(p-q)\oplus\theta$;
11. *if* $\theta\leq p$ *and* $p\neq\theta$, *and* $\alpha> 0$, *then* $\theta\leq\alpha p$ *and* $\alpha p \neq\theta$, *for all* $p,q,r,s,t,w \in X$ *and* $\alpha, \lambda\in\mathbb{R}$.

### Definition 2.6

[4]

Allow $A:X \rightarrow X$ to be a single-valued map.

1. *A* is called a *γ*-order non-extended mapping if there exists a constant $\gamma> 0$ such that
  $$\gamma(p \oplus q) \leq A(p) \oplus A(q),\quad \mbox{for all } p,q \in X; $$
2. *A* is called a strongly comparison map if it is a comparison mapping and $A(p) \propto A(q)$ iff $p \propto q$, for all $p, q \in X$.

### Definition 2.7

[7]

Allow $A: X \rightarrow X$ and $M: X \rightarrow2^{X}$ to be single-valued and set-valued mappings, respectively.

1. *M* is called a weak-comparison map, if for $t_{p} \in M(p)$, $p\propto t_{p}$, and if $p \propto q$, then $\exists \ t_{p} \in M(p)$ and $t_{q} \in M(q)$ such that $t_{p} \propto t_{q} $, for all $p,q \in X$;
2. *M* is called an *α*-weak-non-ordinary difference map associated with *A*, if it is weak comparison and for each $p, q \in X$, $\exists \ \alpha>0 $ and $t_{p} \in M(A(p))$ and $t_{q} \in M(A(q))$ such that
  $$(t_{p}\oplus t_{q})\oplus\alpha\bigl(A(p)\oplus A(q) \bigr)=\theta; $$
3. *M* is called a *λ*-order different weak-comparison map associated with *A* if ∃ a $\lambda> 0$, for all $p,q \in X$ and there exist $t_{p}\in M(A(p))$, $t_{q}\in M(A(q))$ such that
  $$\lambda(t_{p}-t_{q})\propto p-q ; $$
4. *M*, a weak-comparison map, is called an ordered $(\alpha _{A}, \lambda)$-weak-ANODM map, if it is an *α*-weak-non-ordinary difference map and a *λ*-order different weak-comparison map associated with *A*, and $(A+\lambda M)(X)=X$, for $\alpha, \lambda> 0$.

### Definition 2.8

[7]

Let $A: X \rightarrow X$ and $M: X \rightarrow2^{X}$ be a *γ*-order non-extended map and an *α*-non-ordinary difference mapping with respect to *A*, respectively. The resolvent operator $R_{A,\lambda}^{M}: X \rightarrow X$ associated with both *A* and *M* is defined by

1 $$ R_{A, \lambda}^{M} (p) = (A+\lambda M)^{-1} (p),\quad \mbox{for all } p \in X, $$

where $\gamma, \alpha, \lambda>0 $ are constants.

### Definition 2.9

[8]

A map $A: X\times X \rightarrow X$ is called $(\alpha_{1}, \alpha _{2})$-restricted-accretive map, if it is comparison and ∃ constants $0 \leq\alpha_{1}, \alpha_{2} \leq1$ such that

$$\bigl(A(p,\cdot)+I(p)\bigr)\oplus\bigl(A(q, \cdot)+I(q)\bigr) \leq \alpha_{1} \bigl(A(p,\cdot )\oplus A(q,\cdot)\bigr) + \alpha_{2} (p\oplus q),\quad \mbox{for all } p,q \in X, $$

where *I* is the identity map on *X*.

### Lemma 2.4

[7]

*If* $M: X \rightarrow2^{X}$ *and* $A: X \rightarrow X$ *are an* *α*-*weak*-*non*-*ordinary difference map associated with* *A* *and a* *γ*-*order non*-*extended map*, *respectively*, *with* $\alpha{\lambda} \neq1$, *then* $M_{\theta} = \{\theta\oplus p \mid p \in M\}$ *is an* *α*-*weak*-*non*-*ordinary difference map associated with* *A* *and the resolvent operator* $R_{A, \lambda}^{M_{\theta}} = (A+\lambda M_{\theta})^{-1}$ *of* $(A+\lambda M_{\theta})$ *is a single*-*valued for* $\alpha, \lambda>0$, *i*.*e*., $R_{A, \lambda}^{M_{\theta}} : X\rightarrow X$ *of* $M_{\theta}$ *holds*.

### Lemma 2.5

[7]

*Let* $M: X \rightarrow2^{X}$ *and* $A: X \rightarrow X$ *be a* $(\alpha _{A},\lambda)$-*weak*-*ANODD set*-*valued map and a strongly comparison map associated with* $R_{A, \lambda}^{M}$, *respectively*. *Then the resolvent operator* $R_{A, \lambda}^{M}: X \rightarrow X$ *is a comparison map*.

### Lemma 2.6

[7]

*Let* $M: X \rightarrow2^{X}$ *be an ordered* $(\alpha_{A}, \lambda)$-*weak*-*ANODD map and* $A: X \rightarrow X$ *be a* *γ*-*ordered non*-*extended map associated with* $R_{A, \lambda}^{M}$, *for* $\alpha_{A}> \frac {1}{\lambda}$, *respectively*. *Then the following relation holds*:

2 $$ R_{A, \lambda}^{M}(p)\oplus R_{A, \lambda}^{M}(q) \leq\frac{1}{\gamma (\alpha_{A} \lambda-1)} (p \oplus q), \quad \textit{for all } p,q \in X. $$

## Formulation of the problem and existence results

Allow *X* to be a real ordered Banach space and *C* a normal cone having the normal constant $\lambda_{C}$. Let $M,N : X \times X \rightarrow2^{X}$ be set-valued mappings. Suppose $f_{i}, g_{i}: X \rightarrow X $ ($i=1,2$) and $F_{1}, F_{2}: X \times X \rightarrow X$ are single-valued mappings. Now we look at the problem:

For some $(w_{1},w_{2}) \in X \times X$ and $\rho>0$, find $(p,q) \in X\times X$ such that

3 $$ \textstyle\begin{cases} w_{1} \in F_{1}(f_{1}(p), q)+ \rho M(g_{1}(p),q), \\ w_{2} \in F_{2}(p,f_{2}(q)) \oplus N(p,g_{2}(q)). \end{cases} $$

This problem is called a system of generalized implicit ordered variational inclusions (in short SGIOVI). Here, we discuss some special cases of SGIOVI (3).

1. If $\rho=1$, $g_{1}=I$ (the identity mapping on *X*), $f_{2}=I$ and *M* and *N* are single-valued mappings and $M(g_{1}(p),q) = M(q, p)$, then problem (3) reduces to the problem as for $w_{1}, w_{2} \in X$, find $p, q \in X$ such that
  4 $$ \textstyle\begin{cases} w_{1} \leq F_{1}(f_{1}(p), q)+ M(q, p), \\ w_{2} \leq F_{2}(p,q) \oplus N(p,g(q)). \end{cases} $$
  Problem (4) was initiated and studied by [1].
2. If $w_{1}, w_{2}= 0$, $\rho= 1$, $F_{2} = f_{2}= N = g_{2} = 0$, $g_{1}=I$, *M* is a single-valued mapping, then problem (3) is to find $p,q \in X$ such that
  5 $$ 0 \leq F_{1}\bigl(f_{1}(p),q\bigr) + M(q,p). $$
  Problem (5) was initiated and studied by [21].
3. If $w_{2} = 0$, $F_{2}= f_{2}= N = g_{2}=0 $, $g_{1}=I$, $F_{1}(f_{1}(p),q)= f_{1}(p)$ and $M(g_{1}(p), q)= M(p)$, then problem (3) became the problem to find $p \in X$ such that
  6 $$ w_{1} \in f(p)+ \rho M(p). $$
  Problem (6) was initiated and studied by [7].
4. If $\rho, w_{1}= 0$, $F_{1}=f_{1}=g_{1}=M=0$, $f_{2}=g_{2}=I$, $F_{2}(p,f_{2}(q)) = F_{2}(p)$ and $N(p,g_{2}(q))= N(p)$, then problem (3) is converted to the problem of finding $p \in X$ such that
  7 $$ w_{2} \in F_{2}(p) \oplus N(p). $$
  Problem (7) was initiated and studied by [5].
5. If $F_{1}=f_{1}= F_{2} = f_{2} = N = g_{2} = 0$, $w_{2}=0$, $g_{1}=I$ and $M(g_{1}(p),q)= M(p)$, then the problem (3) converted to the problem of finding $p \in X$ such that
  8 $$ w_{1} \in\rho M(p). $$
  Problem (8) was initiated and studied by [3].

Now, we mention the fixed point formulation of SGIOVI (3).

### Lemma 3.1

*The set of elements* $(p,q)\in X \times X$ *become a solution of SGIOVI* (3) *iff* $(p,q)\in X \times X$ *fulfill the relations*:

$$\begin{aligned}& p= R_{A,\lambda}^{M(g_{1}(\cdot),q)} \biggl[A(p)+ \frac{\lambda}{\rho } \bigl(w_{1}-F_{1}\bigl(f_{1}(p),q\bigr)\bigr) \biggr], \\& q= R_{A, \lambda}^{N_{0}(p,g_{2}(\cdot))} \bigl[A(q)+ {\lambda }\bigl(w_{2} \oplus F_{2}\bigl(p,f_{2}(q)\bigr)\bigr) \bigr]. \end{aligned}$$

### Proof

The proof follows from the definition of the resolvent operator (1). □

## Main results

In this section, we present an existence result for the system of generalized implicit ordered variational inclusions (in short SGIOVI), under some apt conditions.

### Theorem 4.1

*Let* *C* *be a normal cone having a normal constant* ${\lambda}_{C}$ *in a real ordered Banach space* *X*. *Let* $A, f_{1}, f_{2}, g_{1}, g_{2} : X \rightarrow X$ *be single*-*valued mappings such that* *A* *is a* ${\lambda}_{A}$-*compression mapping*, $f_{1}$ *is a* ${\lambda}_{f_{1}}$-*compression and* $f_{2}$ *is a* ${\lambda}_{f_{2}}$-*compression and* $g_{1}$, $g_{2}$ *are comparison mappings*, *respectively*. *Let* $F_{1}, F_{2}: X \times X \rightarrow X$ *be single*-*valued mappings such that* $F_{1}$ *is an* $(\alpha_{1}, \alpha_{2})$-*restricted*-*accretive mapping w*.*r*.*t*. $f_{1}$ *and* $F_{2}$ *is an* $(\alpha_{1}', \alpha _{2}')$-*restricted*-*accretive mapping w*.*r*.*t*. $f_{2}$, *respectively*. *Suppose* $M, N_{0} : X \times X \rightarrow2^{X}$ *are the set*-*valued mappings such that* *M* *is a* $(\alpha_{A}, \lambda)$-*weak*-*ANODD set*-*valued mapping and* $N_{0}$ *is a* $(\alpha_{A'}, \lambda)$-*weak*-*ANODD set*-*valued mapping*, *respectively*.

*In addition*, *if* $p_{i}\propto q_{i}$, $R_{A, \lambda_{1}}^{M} (p_{i}) \propto R_{A, \lambda_{1}}^{M} (q_{i})$, $R_{A, \lambda_{2}}^{N_{0}} (p_{i}) \propto R_{A, \lambda_{2}}^{N_{0}} (q_{i})$ *and for all* $\lambda_{1}, \lambda_{2}, \delta_{1}, \delta_{2} > 0$, *the following conditions are satisfied*:

9 $$ \begin{aligned} &R_{A, \lambda_{1}}^{N(p_{1},g_{2}(\cdot))} (q_{1}) \oplus R_{A, \lambda _{2}}^{N(p_{2},g_{2}(\cdot))} (q_{1}) \leq \delta_{1} (p_{1}\oplus p_{2}), \\ &R_{A, \lambda_{1}}^{M(g_{1}(\cdot), q_{1})} (p_{1}) \oplus R_{A, \lambda _{2}}^{M(g_{2}(\cdot), q_{2})} (p_{1}) \leq\delta_{2} (q_{1}\oplus q_{2}), \end{aligned} $$

*and*

10 $$ \begin{aligned} &\lambda_{C}( \lambda_{A}\mu_{1}+\lambda_{\alpha_{1}'}\mu_{2}+ \delta _{2}) < 1 - \biggl(\frac{\lambda_{C}\mu_{1}\lambda_{\alpha_{1}}\lambda _{f_{1}}}{\rho} \biggr), \\ &\lambda_{C}(\lambda_{A'}\mu_{2} + \lambda_{\alpha_{2}}\lambda _{f_{2}}\lambda_{\alpha_{2}'}+ \delta_{1}) < 1- \biggl(\frac{\lambda _{C}\mu_{1}\lambda_{\alpha_{2}}}{\rho} \biggr). \end{aligned} $$

*Then the SGIOVI* (3) *grants a solution* $(p,q)\in X\times X$.

### Proof

By Lemma 2.6, we know that the resolvent operator $R_{A, \lambda }^{M}(\cdot)$ and $R_{A, \lambda}^{N_{0}}(\cdot)$ are $\mu_{1}$-Lipschitz continuous and $\mu_{2}$-Lipschitz continuous, respectively.

Here $\mu_{1}= \frac{1}{\gamma_{1}(\alpha_{A}\lambda-1)}$ and $\mu _{2}=\frac{1}{\gamma_{2}(\alpha_{A'}\lambda-1)}$.

Now, define a map $G: X \times X \rightarrow X \times X$ by

11 $$ G(p,q)= \bigl(T(p,q),S(p,q)\bigr), \quad \forall (p,q) \in X \times X, $$

where $T: X \times X \rightarrow X$ and $S: X \times X \rightarrow X$ are the mappings defined as

12 $$\begin{aligned}& T(p,q)= R_{A, \lambda}^{M(g_{1}(\cdot),q)} \biggl[A + \frac{\lambda}{\rho } \bigl(w_{1}-F_{1}\bigl(f_{1}( \cdot),q\bigr)\bigr) \biggr](p), \end{aligned}$$

13 $$\begin{aligned}& S(p,q)= R_{A, \lambda}^{N_{0}(p,g_{2}(\cdot))} \bigl[A + \lambda \bigl(w_{2} \oplus F_{2}\bigl(p,f_{2}(\cdot) \bigr)\bigr) \bigr](q). \end{aligned}$$

For any $p_{i}, q_{i} \in X$ and $p_{i} \propto q_{j}$ ($i,j = 1,2$). By using (12), Definition 2.5, Definition 2.9, Lemma 2.6 and Lemma 2.3, we have

14 $$\begin{aligned} 0 \leq& T(p_{1},q_{1}) \oplus T(p_{2},q_{2}) \\ =& R_{A, \lambda}^{M(g_{1}(\cdot),q_{1})} \biggl[A + \frac{\lambda}{\rho } \bigl(w_{1}-F_{1}\bigl(f_{1}( \cdot),q_{1}\bigr)\bigr) \biggr](p_{1}) \\ &{}\oplus R_{A, \lambda}^{M(g_{1}(\cdot),q_{2})} \biggl[A + \frac{\lambda }{\rho} \bigl(w_{1}-F_{1}\bigl(f_{1}( \cdot),q_{2}\bigr)\bigr) \biggr](p_{2}) \\ \leq& R_{A, \lambda}^{M(g_{1}(\cdot),q_{1})} \biggl[A + \frac{\lambda }{\rho} \bigl(w_{1}-F_{1}\bigl(f_{1}( \cdot),q_{1}\bigr)\bigr) \biggr](p_{1}) \\ &{}\oplus R_{A, \lambda}^{M(g_{1}(\cdot),q_{1})} \biggl[A + \frac{\lambda }{\rho} \bigl(w_{1}-F_{1}\bigl(f_{1}( \cdot),q_{2}\bigr)\bigr) \biggr](p_{2}) \\ &{}\oplus R_{A, \lambda}^{M(g_{1}(\cdot),q_{1})} \biggl[A + \frac{\lambda }{\rho} \bigl(w_{1}-F_{1}\bigl(f_{1}( \cdot),q_{2}\bigr)\bigr) \biggr](p_{2}) \\ &{}\oplus R_{A, \lambda}^{M(g_{1}(\cdot),q_{2})} \biggl[A + \frac{\lambda }{\rho} \bigl(w_{1}-F_{1}\bigl(f_{1}( \cdot),q_{2}\bigr)\bigr) \biggr](p_{2}) \\ \leq& \mu_{1} \biggl[ A(p_{1})\oplus A(p_{2}) + \frac{\lambda}{\rho} \bigl(F_{1}\bigl(f_{1}(p_{1}),q_{1} \bigr)\oplus F_{1}\bigl(f_{1}(p_{2}),q_{2} \bigr) \bigr) \biggr] \\ &{}\oplus\delta_{2}(q_{1}\oplus q_{2}) \\ \leq&\mu_{1} \biggl[\lambda_{A} (p_{1}\oplus q_{2}) + \frac{\lambda }{\rho} \bigl(\alpha_{1} \bigl(f_{1}(p_{1})\oplus f_{2}(p_{2}) \bigr) + \alpha_{2} (q_{1}\oplus q_{2}) \bigr) \biggr] \\ &{}\oplus\delta_{2}(q_{1}\oplus q_{2}) \\ \leq& \mu_{1} \biggl[\lambda_{A}(p_{1}\oplus p_{2})+ \frac{\lambda}{\rho } \bigl(\alpha_{1} \lambda_{f_{1}} (p_{1}\oplus p_{2})+\alpha _{2}(q_{1}\oplus q_{2}) \bigr) \biggr] \\ &{}\oplus\delta_{2}(q_{1}\oplus q_{2}) \\ =& \mu_{1} \biggl[ \biggl( \lambda_{A} + \frac{\lambda\alpha_{1} \lambda _{f_{1}}}{\rho} \biggr) (p_{1}\oplus p_{2}) + \frac{\lambda\alpha _{2}}{\rho} (q_{1}\oplus q_{2}) \biggr] \\ &{}\oplus\delta_{2}(q_{1}\oplus q_{2}) \\ \leq& \biggl[\mu_{1} \biggl(\frac{\lambda_{A} \rho+ \lambda\alpha_{1} \lambda_{f_{1}}}{\rho} \biggr) (p_{1}\oplus p_{2}) + \biggl(\frac{\mu_{1} \lambda\alpha_{1}}{\rho} \biggr) (q_{1}\oplus q_{2}) \biggr] \\ &{}\oplus\delta_{2}(q_{1}\oplus q_{2}) \\ =& \biggl[\mu_{1} \biggl(\frac{\lambda_{A} \rho+ \lambda\alpha_{1} \lambda_{f_{1}}}{\rho} \biggr) (p_{1}\oplus p_{2}) + \biggl(\frac{\mu_{1} \lambda\alpha_{1}}{\rho} \biggr) (q_{1}\oplus q_{2}) \biggr] \\ &{}\oplus\delta_{2}(q_{1}\oplus q_{2}). \end{aligned}$$

By Definition 2.2 and Lemma 2.2, we have

$$\begin{aligned}& \bigl\Vert T(p_{1},q_{1})\oplus T(p_{2},q_{2}) \bigr\Vert = \bigl\Vert T(p_{1},q_{1})- T(p_{2},q_{2}) \bigr\Vert \\ & \hphantom{ \bigl\Vert T(p_{1},q_{1})\oplus T(p_{2},q_{2}) \bigr\Vert }\leq \lambda_{C} \biggl\Vert \biggl[ \mu_{1} \biggl(\frac{\lambda_{A} \rho+ \lambda\alpha_{1} \lambda_{f_{1}}}{\rho} \biggr) (p_{1}\oplus p_{2}) \\ & \hphantom{ \bigl\Vert T(p_{1},q_{1})\oplus T(p_{2},q_{2}) \bigr\Vert ={}}{}+ \biggl(\frac{\mu_{1} \lambda\alpha_{1}}{\rho} \biggr) (q_{1}\oplus q_{2})\biggr] \oplus\delta_{1}(q_{1}\oplus q_{2}) \biggr\Vert \\ & \hphantom{ \bigl\Vert T(p_{1},q_{1})\oplus T(p_{2},q_{2}) \bigr\Vert }= \lambda_{C} \biggl\Vert \biggl[\mu_{1} \biggl(\frac{\lambda_{A} \rho+ \lambda \alpha_{1} \lambda_{f_{1}}}{\rho} \biggr) (p_{1}\oplus p_{2}) \\ & \hphantom{ \bigl\Vert T(p_{1},q_{1})\oplus T(p_{2},q_{2}) \bigr\Vert ={}}{}+ \biggl(\frac{\mu_{1} \lambda\alpha_{1}}{\rho} \biggr) (q_{1}\oplus q_{2})\biggr]- \delta_{1}(q_{1}\oplus q_{2}) \biggr\Vert , \\ & \bigl\Vert T(p_{1},q_{1})\oplus T(p_{2},q_{2}) \bigr\Vert \leq \lambda_{C} \biggl\Vert \biggl[\mu _{1} \biggl(\frac{\lambda_{A} \rho+ \lambda\alpha_{1} \lambda _{f_{1}}}{\rho} \biggr) (p_{1}\oplus p_{2}) \\ & \hphantom{ \bigl\Vert T(p_{1},q_{1})\oplus T(p_{2},q_{2}) \bigr\Vert \leq{}}{}+ \biggl(\frac{\mu_{1} \lambda\alpha_{1}}{\rho} \biggr) (q_{1}\oplus q_{2})\biggr] \biggr\Vert +\lambda_{C} \delta_{1} \bigl( \Vert q_{1} \oplus q_{2} \Vert \bigr) \\ & \hphantom{ \bigl\Vert T(p_{1},q_{1})\oplus T(p_{2},q_{2}) \bigr\Vert }\leq \lambda_{C}\mu_{1} \biggl( \frac{\lambda_{A} \rho+ \lambda\alpha _{1} \lambda_{f_{1}}}{\rho} \biggr) \Vert p_{1}-p_{2} \Vert \\ & \hphantom{ \bigl\Vert T(p_{1},q_{1})\oplus T(p_{2},q_{2}) \bigr\Vert \leq{}}{}+\lambda_{C} \biggl(\frac{\mu_{1}\lambda\alpha_{2}+\delta_{1}\rho}{\rho } \biggr) \Vert q_{1}-q_{2} \Vert . \end{aligned}$$

That is,

15 $$\begin{aligned} \bigl\Vert T(p_{1},q_{1})- T(p_{2},q_{2}) \bigr\Vert \leq& \lambda_{C} \mu_{1} \biggl(\frac {\lambda_{A} \rho+ \lambda\alpha_{1} \lambda_{f_{1}}}{\rho} \biggr) \Vert p_{1}-p_{2} \Vert \\ &{} + \lambda_{C} \biggl(\frac{\mu_{1}\lambda\alpha_{2}+\delta_{1}\rho }{\rho} \biggr) \Vert q_{1}-q_{2} \Vert . \end{aligned}$$

For any $p_{i}, q_{j} \in X$, $p_{i}\propto q_{j}$ ($i,j=1,2$), and by using (13), Definition 2.5, Definition 2.6, Lemma 2.3 and Lemma 2.6, we have

16 $$\begin{aligned} 0 \leq& S(p_{1},q_{1}) \oplus S(p_{2},q_{2}) \\ =& \bigl(R_{A, \lambda}^{N_{0}(p_{1},g_{2}(\cdot))} \bigl[A+ {\lambda } \bigl(w_{2}\oplus F_{2}\bigl(p_{1},f_{2}( \cdot)\bigr)\bigr) \bigr](q_{1}) \\ &{} \oplus R_{A, \lambda}^{N_{0}(p_{2},g_{2}(\cdot))} \bigl[A+ {\lambda } \bigl(w_{2}\oplus F_{2}\bigl(p_{2},f_{2}( \cdot)\bigr)\bigr) \bigr](q_{2}) \bigr) \\ \leq& \bigl(R_{A, \lambda}^{N_{0}(p_{1},g_{2}(\cdot))} \bigl[A+ {\lambda } \bigl(w_{2}\oplus F_{2}\bigl(p_{1},f_{2}( \cdot)\bigr)\bigr) \bigr](q_{1}) \\ &{}\oplus R_{A, \lambda}^{N_{0}(p_{1},g_{2}(\cdot))} \bigl[A+ {\lambda } \bigl(w_{2}\oplus F_{2}\bigl(p_{2},f_{2}( \cdot)\bigr)\bigr) \bigr](q_{2}) \bigr) \\ &{}\oplus \bigl(R_{A, \lambda}^{N_{0}(p_{1},g_{2}(\cdot))} \bigl[A+ {\lambda} \bigl(w_{2}\oplus F_{2}\bigl(p_{2},f_{2}( \cdot)\bigr)\bigr) \bigr](q_{2}) \\ &{}\oplus R_{A, \lambda}^{N_{0}(p_{2},g_{2}(\cdot))} \bigl[A+ {\lambda } \bigl(w_{2}\oplus F_{2}\bigl(p_{2},f_{2}( \cdot)\bigr)\bigr) \bigr](q_{2}) \bigr) \\ \leq& \mu_{2} \bigl[ \bigl(A(q_{1})+ {\lambda} \bigl(w_{2}\oplus F_{2}\bigl(p_{1},f_{2}(q_{1}) \bigr)\bigr) \bigr) \\ &{}\oplus\bigl(A(q_{2})+ {\lambda}\bigl(w_{2}\oplus F_{2}\bigl(p_{2},f_{2}(q_{2})\bigr) \bigr)\bigr) \bigr] \oplus\delta_{1} (p_{1}\oplus p_{2}) \\ \leq& \mu_{2} \bigl[A(q_{1})\oplus A(q_{2}) + \lambda \bigl(F_{2}\bigl(p_{1},f_{2}(q_{1}) \bigr)\oplus F_{2}\bigl(p_{2},f_{2}(q_{2}) \bigr) \bigr) \bigr] \\ &{} \oplus\delta_{1}(p_{1}\oplus p_{2}) \\ \leq& \mu_{2} \bigl[\lambda_{A}(q_{1}\oplus q_{2}) + \lambda\bigl(\alpha _{1}'(p_{1} \oplus p_{2}) +\alpha_{2}'\lambda_{f_{2}} (q_{1}\oplus q_{2})\bigr) \bigr]\oplus\delta_{1}(p_{1} \oplus p_{2}) \\ =& \mu_{2} \bigl[\bigl(\lambda_{A}+\lambda \alpha_{2}'\lambda _{f_{2}}\bigr) (q_{1} \oplus q_{2}) + \lambda\alpha_{1}'(p_{1} \oplus p_{2}) \bigr]\oplus\delta_{1}(p_{1}\oplus p_{2}). \end{aligned}$$

By Definition 2.2 and Lemma 2.2, we have

$$\begin{aligned} \bigl\Vert S(p_{1},q_{1}) \oplus S(p_{2},q_{2}) \bigr\Vert =& \bigl\Vert S(p_{1},q_{1})-S(p_{2},q_{2}) \bigr\Vert \\ \leq& \lambda_{C} \bigl\Vert \bigl[\mu_{2} \lambda_{A}+\lambda\alpha _{2}' \lambda_{f_{2}}(q_{1}\oplus q_{2}) + \mu_{2}\lambda\alpha _{1}'(p_{1} \oplus p_{2}) \bigr] \\ &{}\oplus\delta_{2}(p_{1}\oplus p_{2}) \bigr\Vert \\ \leq& \lambda_{C} \bigl\Vert \mu_{2} \lambda_{A}+\lambda\alpha _{2}' \lambda_{f_{2}}(q_{1}\oplus q_{2}) + \mu_{2}\lambda\alpha _{1}'(p_{1} \oplus p_{2}) \bigr\Vert \\ &{}+ \lambda_{C}\delta_{2} \Vert p_{1}- p_{2} \Vert \\ \leq& \lambda_{C} \mu_{2} \bigl(\lambda_{A}+ \lambda\alpha_{2}' \lambda _{f_{2}}\bigr) \Vert q_{1}- q_{2} \Vert \\ &{} + \lambda_{C} \bigl(\mu_{2} \lambda \alpha_{1}' +\delta_{2}\bigr) \Vert p_{1}- p_{2} \Vert . \end{aligned}$$

That is,

17 $$\begin{aligned} \bigl\Vert S(p_{1},q_{1})-S(p_{2},q_{2}) \bigr\Vert \leq& \lambda_{C} \mu_{2} \bigl(\lambda _{A}+\lambda\alpha_{2}' \lambda_{f_{2}} \bigr) \Vert q_{1}- q_{2} \Vert \\ &{}+ \lambda_{C} \bigl(\mu_{2} \lambda \alpha_{1}' +\delta_{2}\bigr) \Vert p_{1}- p_{2} \Vert . \end{aligned}$$

From (15) and (17), we have

18 $$\begin{aligned} \bigl\Vert T(p_{1},q_{1})- T(p_{2},q_{2}) \bigr\Vert +& \bigl\Vert S(p_{1},q_{1})-S(p_{2},q_{2}) \bigr\Vert \\ \leq& \lambda_{C}\mu_{1} \biggl(\frac{\lambda_{A} \rho+ \lambda\alpha _{1} \lambda_{f_{1}}}{\rho} \biggr) \Vert p_{1}-p_{2} \Vert \\ &{}+ \lambda_{C} \biggl(\frac{\mu_{1}\lambda\alpha_{2}+\delta_{1}\rho }{\rho} \biggr) \Vert q_{1}-q_{2} \Vert \\ &{}+ \lambda_{C} \mu_{2} \bigl(\lambda_{A'}+ \lambda\alpha_{2}' \lambda _{f_{2}}\bigr) \Vert q_{1}- q_{2} \Vert \\ &{} + \lambda_{C} \bigl(\mu_{2} \lambda \alpha_{1}' +\delta_{2}\bigr) \Vert p_{1}- p_{2} \Vert \\ =& \biggl[ \biggl(\frac{\lambda_{A} \rho+ \lambda\alpha_{1} \lambda _{f_{1}}}{\rho} \biggr) \\ &{}+\lambda_{C} \bigl(\mu_{2} \lambda \alpha_{1}' +\delta_{2}\bigr) \biggr] \Vert p_{1}- p_{2} \Vert \\ &{}+ \biggl[\lambda_{C} \biggl(\frac{\mu_{1}\lambda\alpha_{2}+\delta _{1}\rho}{\rho} \biggr) \\ &{}+\lambda_{C} \mu_{2} \bigl(\lambda_{A'}+ \lambda\alpha_{2}' \lambda _{f_{2}}\bigr) \biggr] \Vert q_{1}-q_{2} \Vert \\ =& \Omega_{1} \Vert p_{1}- p_{2} \Vert + \Omega_{2} \Vert p_{1}- p_{2} \Vert \\ \leq& \max\{\Omega_{1}, \Omega_{2}\} \bigl( \Vert p_{1}- p_{2} \Vert + \Vert q_{1}- q_{2} \Vert \bigr), \end{aligned}$$

where

$$\Omega_{1} = \biggl[\lambda_{C} \biggl(\frac{\lambda_{A} \rho+ \lambda \alpha_{1} \lambda_{f_{1}}}{\rho} \biggr)+\lambda_{C} \bigl(\mu_{2} \lambda \alpha_{1}' +\delta_{2}\bigr) \biggr] $$

and

$$\Omega_{2} = \biggl[\lambda_{C} \biggl(\frac{\mu_{1}\lambda\alpha _{2}+\delta_{1}\rho}{\rho} \biggr)+\lambda_{C} \mu_{2} \bigl(\lambda _{A'}+ \lambda\alpha_{2}' \lambda_{f_{2}}\bigr) \biggr]. $$

Now, we define $\|(p,q)\|_{\ast}$ on $X \times X$ by

19 $$ \bigl\Vert (p,q) \bigr\Vert _{\ast} = \Vert p \Vert + \Vert q \Vert , \quad \forall(p,q)\in X \times X. $$

One can easily show that $(X\times X, \|\cdot\|_{\ast})$ is a Banach space. Hence from (11), (18) and (19), we have

20 $$ \bigl\Vert G(p_{1},q_{1})-G(p_{2},q_{2}) \bigr\Vert _{\ast}\leq\max\{\Omega_{1},\Omega _{2}\}\bigl( \Vert p_{1}- p_{2} \Vert + \Vert q_{1}- q_{2} \Vert \bigr). $$

By (10), we know that $\max\{\Omega_{1},\Omega_{2}\} < 1$. It follows from (20) that *G* is a contraction. Hence ∃ unique $(p,q)\in X \times X$ such that

$$G(p,q)= (p,q). $$

This leads to

$$p= R_{A, \lambda}^{M(g_{1}(\cdot),q)} \biggl[A+\frac{{\lambda}}{\rho} \bigl(w_{1}-F_{1}\bigl(f_{1}(\cdot),q\bigr)\bigr) \biggr](p) $$

and

$$q=R_{A, \lambda}^{N_{0}(p,g_{2}(\cdot))} \bigl[A+ {\lambda}\bigl(w_{2}\oplus F_{2}\bigl(p, f_{2}(\cdot)\bigr)\bigr) \bigr](q). $$

It is determined by Lemma 3.1 that $(p,q)$ is a solution of SGIOVI (3). □

## Convergence analysis and iterative algorithm

This part of the article is associated with the construction of an iterative scheme as well as the strong convergence of the sequences achieved by the said scheme to the exact solution of SGIOVI (3).

Allow *C* to be a normal cone with the normal constant $\lambda_{C}$ in a real ordered Banach space *X*. Let $M: X\times X \rightarrow2^{X}$ and $N:X\times X \rightarrow2^{X}$ be set-valued maps. Assume that $f_{1}, f_{2},g_{1}, g_{2}: X \rightarrow X$ and $F_{1},F_{2}: X \times X \rightarrow X$ are single-valued maps.

For the initial guess $(p_{0},q_{0}) \in X \times X$, assume that $p_{0} \propto p_{1} $, $q_{0} \propto q_{1}$. We define an iterative sequence $\{(p_{n},q_{n})\}$ and let $p_{n+1}\propto p_{n}$, $q_{n+1}\propto q_{n}$ such that

21 $$\begin{aligned}& p_{n+1} = \alpha_{n}p_{n} + (1- \alpha_{n}) R_{A, \lambda }^{M(g_{1}(\cdot),q_{n})} \biggl[A(\cdot)+ \frac{{\lambda}}{\rho} \bigl(w_{1}-F_{1}\bigl(f_{1}( \cdot),q_{n}\bigr)\bigr) \biggr] (p_{n}), \end{aligned}$$

22 $$\begin{aligned}& q_{n+1} = \alpha_{n}q_{n}+ (1- \alpha_{n})R_{A, \lambda }^{N_{0}(p_{n},g_{2}(\cdot))} \bigl[A(\cdot)+ {\lambda} \bigl(w_{2}\oplus F_{2}\bigl(p_{n}, g_{2}(\cdot)\bigr)\bigr) \bigr](q_{n}). \end{aligned}$$

For $n= 0,1,2,3, \ldots$ , where $0 \leq\alpha_{n} < 1$ with $\limsup_{n} \alpha_{n} < 1$.

### Lemma 5.1

[17]

*Allow* $\{\vartheta_{n}\}$ *and* $\varsigma_{n}$ *to be sequences of nonnegative real numbers such that they satisfy*

(i) $0 \leq\varsigma_{n} < 1$, $n= 0,1,2,\ldots$ *and* $\limsup_{n} \varsigma_{n} <1$;
(ii) $\vartheta_{n+1} \leq\varsigma_{n} \vartheta_{n}$, $n=0,1,2, \ldots$ .

*Then* $\{\vartheta_{n}\}$ *approaches zero as* *n* *moves to* ∞.

### Theorem 5.2

*Allow* *X*, *C*, *M*, *N*, $N_{0}$, $f_{1}$, $f_{2}$, $g_{1}$, $g_{2}$, $F_{1}$ *and* $F_{2}$ *to be as in Theorem* 4.1 *such that all the assertions of Theorem* 4.1 *are valid*. *Then the sequence* $\{(p_{n}, q_{n})\}$ *formulated by Algorithm* (21) *and* (22) *converges strongly to the unique solution* $\{(p,q)\}$ *of SGIOVI* (3).

### Proof

By Theorem 4.1, the SGIOVI (3) admits a unique solution $(p,q)$. It follows from Lemma 3.1 that

23 $$ p = \alpha_{n}p + (1-\alpha_{n}) R_{A, \lambda}^{M(g_{1}(\cdot),q)} \bigl[A(\cdot)+{\lambda}\bigl(w_{1}-F_{1} \bigl(f_{1}(\cdot),q\bigr)\bigr) \bigr](p) $$

and

24 $$ q = \alpha_{n}q+ (1-\alpha_{n})R_{A, \lambda}^{N_{0}(p,g_{2}(\cdot ))} \bigl[A(\cdot)+ {\lambda}\bigl(w_{2}\oplus F_{2}\bigl(p, g_{2}(\cdot)\bigr)\bigr) \bigr](q). $$

By (21), (23) and Lemma 2.3, we get

25 $$\begin{aligned} 0 \leq& p_{n+1} \oplus p \\ =& \alpha_{n}p_{n} + (1-\alpha_{n}) R_{A, \lambda}^{M(g_{1}(\cdot ),q_{n})} \bigl[A(\cdot)+{\lambda}\bigl(w_{1}-F_{1} \bigl(f_{1}(\cdot),q_{n}\bigr)\bigr) \bigr]p_{n} \\ &{}\oplus\alpha_{n}p + (1-\alpha_{n}) R_{A, \lambda}^{M(g_{1}(\cdot ),q)} \bigl[A(\cdot)+{\lambda}\bigl(w_{1}-F_{1} \bigl(f_{1}(\cdot),q\bigr)\bigr) \bigr](p) \\ =& \alpha_{n}(p_{n}\oplus p) + (1-\alpha_{n}) \bigl[R_{A, \lambda }^{M(g_{1}(\cdot),q_{n})} \bigl[A(p_{n})+{\lambda } \bigl(w_{1}-F_{1}\bigl(f_{1}(p_{n}),q_{n} \bigr)\bigr) \bigr] \\ &{}\oplus R_{A, \lambda}^{M(g_{1}(\cdot),q)} \bigl[A(p)+{\lambda } \bigl(w_{1}-F_{1}\bigl(f_{1}(p),q\bigr)\bigr) \bigr] \bigr]. \end{aligned}$$

By using the same argument as in Theorem 4.1, for (14), we have

26 $$\begin{aligned} \Vert p_{n+1} \oplus p \Vert =& \Vert p_{n+1}-p \Vert \\ \leq& \alpha_{n} \Vert p_{n}-p \Vert +(1- \alpha_{n}) \biggl[\frac{\lambda_{C} \mu _{1}(\lambda_{A}\rho+ \lambda\alpha_{1}\lambda_{f_{1}})}{\rho} \Vert p_{n}-p \Vert \\ &{}+ \frac{\lambda_{C}(\mu_{1}\lambda\alpha_{2}+ \delta_{1}\rho)}{\rho } \Vert q_{n}-q \Vert \biggr] \\ =& \biggl(\alpha_{n} + \frac{(1-\alpha_{n}) \lambda_{C} \mu_{1}(\lambda _{A}\rho+ \lambda\alpha_{1}\lambda_{f_{1}})}{\rho} \biggr) \Vert p_{n}-p \Vert \\ &{}+ \biggl(\frac{\lambda_{C}(1-\alpha_{n})(\mu_{1}\lambda\alpha_{2}+ \delta_{1}\rho)}{\rho} \biggr) \Vert q_{n}-q \Vert . \end{aligned}$$

Similarly, it follows from (22) and (24) that

27 $$\begin{aligned} 0 \leq& q_{n+1}\oplus q_{n} \\ =& \bigl(\alpha_{n}q_{n}+ (1-\alpha_{n})R_{A, \lambda }^{N_{0}(p_{n},g_{2}(\cdot))} \bigl[A(q_{n})+ {\lambda}\bigl(w_{2}\oplus F_{2} \bigl(p_{n}, g_{2}(q_{n})\bigr)\bigr) \bigr] \\ &{}\oplus\alpha_{n}q+ (1-\alpha_{n})R_{A, \lambda}^{N_{0}(p,g_{2}(\cdot ))} \bigl[A(q)+ {\lambda}\bigl(w_{2}\oplus F_{2} \bigl(p,g_{2}(q)\bigr)\bigr) \bigr] \bigr) \\ \leq& \alpha_{n} (q_{n}\oplus q) + (1- \alpha_{n}) \bigl(R_{A, \lambda }^{N_{0}(p_{n},g_{2}(\cdot))} \bigl[A(q_{n})+ {\lambda}\bigl(w_{2}\oplus F_{2}\bigl(p_{n}, g_{2}(q_{n})\bigr)\bigr) \bigr] \\ &{}\oplus R_{A, \lambda}^{N_{0}(p,g_{2}(\cdot))} \bigl[A(q)+ {\lambda } \bigl(w_{2}\oplus F_{2}\bigl(p,g_{2}(q)\bigr) \bigr) \bigr] \bigr). \end{aligned}$$

Importing the same logic as in Theorem 4.1 for (16), we have

28 $$\begin{aligned} \Vert q_{n+1} \oplus q \Vert =& \Vert q_{n+1}-q \Vert \\ \leq& \bigl(\alpha_{n} + \lambda_{C}\mu_{2} \bigl(\lambda_{A^{\prime}}+ \lambda \alpha_{2}' \lambda_{f_{2}}\bigr) \Vert q_{n}-q \Vert \\ &{}+ \lambda_{C}(1-\alpha_{n}) \bigl(\bigl( \mu_{2}\lambda\alpha_{1}'+\delta _{2}\bigr)\bigr) \Vert p_{n}-p \Vert \bigr). \end{aligned}$$

From (26) and (28) we have

29 $$\begin{aligned} \Vert p_{n+1}-p \Vert + \Vert q_{n+1}-q \Vert \leq& \biggl[ \biggl(\alpha_{n}+ \frac {(1-\alpha_{n})\lambda_{C}\mu_{1}(\lambda_{A}\rho+\lambda\alpha _{1}\lambda_{f_{2}})}{\rho} \biggr) \Vert p_{n}-p \Vert \biggr] \\ &{}+ \biggl(\frac{\lambda_{C}(1-\alpha_{n})(\mu_{1}\lambda\alpha _{2}+\delta_{1}\rho)}{\rho} \biggr) \Vert q_{n}-q \Vert \\ &{}+ \bigl(\alpha_{n}+\mu_{2}\lambda_{C}\bigl( \lambda_{A^{\prime}}+\lambda\alpha _{2}' \lambda_{f_{2}}\bigr) \bigr) \Vert q_{n}-q \Vert \\ &{}+\lambda_{C} (1-\alpha_{n}) \bigl(\mu_{2} \lambda\alpha_{n}'+\delta _{2}\bigr) \Vert p_{n}-p \Vert \\ =&\alpha_{n} \bigl( \Vert p_{n}-p \Vert + \Vert q_{n}-q \Vert \bigr) \\ &{}+(1-\alpha_{n}) \biggl[ \biggl(\frac{\lambda_{C}\mu_{1}(\lambda_{A}\rho +\lambda\alpha_{1}\lambda_{f_{1}})}{\rho} \\ &{}+ \lambda_{C}\bigl(\mu_{2}+\lambda \alpha_{1}'\lambda\bigr) \biggr) \biggr] \Vert p_{n}-p \Vert \\ &{}+(1-\alpha_{n}) \biggl[ \biggl(\frac{\lambda_{C}(\mu_{1}\lambda\alpha _{2}+\delta_{1}\rho)}{\rho} \\ &{}+\lambda_{C}\mu_{2}\bigl(\lambda_{A^{\prime}}+ \lambda\alpha_{2}'\lambda _{f_{2}}\bigr) \biggr) \Vert q_{n}-q \Vert \biggr] \\ =& \alpha_{n}\bigl( \Vert p_{n}-p \Vert + \Vert q_{n}-q \Vert \bigr) \\ &{}+(1-\alpha_{n}) \bigl(\Omega_{1} \Vert p_{n}-p \Vert +\Omega_{2} \Vert q_{n}-q \Vert \bigr). \end{aligned}$$

By (10), we know that $\max\{\Omega_{1}, \Omega_{2}\} < 1$. Then (29) becomes

30 $$\begin{aligned} \Vert p_{n+1}-p \Vert + \Vert q_{n+1}-q \Vert \leq&\alpha_{n}\bigl( \Vert p_{n}-p \Vert + \Vert q_{n}-q \Vert \bigr) \\ &{}+(1-\alpha_{n})\Omega\bigl( \Vert p_{n}-p \Vert + \Vert q_{n}-q \Vert \bigr), \end{aligned}$$

where $\Omega=\max\{\Omega_{1},\Omega_{2}\}$ and $\Omega_{1}= [\lambda_{C} (\frac{\lambda_{A} \rho+ \lambda\alpha_{1} \lambda _{f_{1}}}{\rho} )+\lambda_{C} (\mu_{2} \lambda\alpha_{1}' +\delta_{2}) ]$, $\Omega_{2}= [\lambda_{C} (\frac{\mu_{1}\lambda\alpha _{2}+\delta_{1}\rho}{\rho} )+\lambda_{C} \mu_{2} (\lambda _{A'}+\lambda\alpha_{2}' \lambda_{f_{2}}) ]$. Let $\vartheta_{n}= (\|p_{n}-p\|+\|q_{n}-q\| )$ and $\varsigma _{n}=\Omega+(1-\Omega)\alpha_{n}$, then (30) can be rewritten as

$$\vartheta_{n+1}\leq\varsigma_{n}\vartheta_{n}, \quad n=0,1,2,\ldots. $$

Choosing $\varsigma_{n}$, we know that $\limsup_{n} \varsigma_{n}<1$. It follows from Lemma 5.1 that $0\leq\varsigma_{n}\leq1$. Therefore, $\{(p_{n},q_{n})\}$ converge strongly to the unique solution $\{(p,q)\}$ of the SGIOVI (3). □

## Conclusion

System of generalized ordered variational inclusions can be viewed as natural and innovative generalizations of the system of generalized ordered variational inequalities. Two of the most difficult and important problems related to inclusions are the establishment of generalized inclusions and the development of an iterative algorithm. In this article, a system of generalized ordered variational inclusions is introduced and studied which is more general than many existing systems of ordered variational inclusions in the literature. An iterative algorithm is established with the ⊕ operator to approximate the solution of our system, and a convergence criterion is also discussed.

We remark that our results are new and useful for further research and one can extend these results in higher dimensional spaces. Much more work is needed in all these areas to develop a sound basis for applications of the system of general ordered variational inclusions in engineering and physical sciences.
